# Real-world clinical outcomes of treatment with molnupiravir for patients with mild-to-moderate coronavirus disease 2019 during the Omicron variant pandemic

**DOI:** 10.1007/s10238-022-00949-3

**Published:** 2022-12-05

**Authors:** Yasuhito Suzuki, Yoko Shibata, Hiroyuki Minemura, Takefumi Nikaido, Yoshinori Tanino, Atsuro Fukuhara, Ryuzo Kanno, Hiroyuki Saito, Shuzo Suzuki, Yayoi Inokoshi, Eiichiro Sando, Hirofumi Sakuma, Tatsuho Kobayashi, Hiroaki Kume, Masahiro Kamimoto, Hideko Aoki, Akira Takama, Taku Iizuka, Takamichi Kamiyama, Masaru Nakayama, Kiyoshi Saito, Koichi Tanigawa, Masahiko Sato, Yuichi Waragai, Toshiyuki Kambe, Norio Kanzaki, Teruhisa Azuma, Hiromasa Okamoto, Keiji Sakamoto, Yuichi Nakamura, Hiroshi Ohtani, Mitsuru Waragai, Shinsaku Maeda, Tokiya Ishida, Keishi Sugino, Wataru Abe, Yasuhiko Tsukada, Tomoyoshi Lee, Ryuki Yamada, Riko Sato, Takumi Onuma, Hikaru Tomita, Mikako Saito, Natsumi Watanabe, Mami Rikimaru, Takaya Kawamata, Julia Morimoto, Ryuichi Togawa, Yuki Sato, Junpei Saito, Kenya Kanazawa, Sugihiro Hamaguchi, Ken Iseki

**Affiliations:** 1 Department of Pulmonary Medicine, School of Medicine, Fukushima Medical University, 1 Hikarigaoka, Fukushima City, Fukushima Prefecture 960-1295 Japan; 2 Department of Pulmonary Medicine, Ohara General Hospital, Fukushima, Japan; 3 Department of Thoracic Surgery, Fukushima Red Cross Hospital, Fukushima, Japan; 4 Department of Internal Medicine, Fujita General Hospital, Fukushima, Japan; 5 Department of Pulmonary Medicine, Saiseikai Fukushima General Hospital, Fukushima, Japan; 6 Department of General Internal Medicine and Clinical Infectious Diseases, Fukushima Medical University, Fukushima, Japan; 7 Department of Internal Medicine, Saiseikai Kawamata Hospital, Fukushima, Japan; 8 Department of Emergency and Critical Care Medicine, Aizu Chuo Hospital, Fukushima, Japan; 9 Department of Infectious Disease and Respiratory Medicine, Aizu Medical Center, Fukushima Medical University, Fukushima, Japan; 10 Department of Internal Medicine, Takeda General Hospital, Fukushima, Japan; 11 Department of Pediatric Medicine, Bange Kousei General Hospital, Fukushima, Japan; 12 Department of Surgery, Yurin Hospital, Fukushima, Japan; 13 Department of Internal Medicine, Yurin Hospital, Fukushima, Japan; 14 Department of Pediatric Surgery, Iwaki City Medical Center, Fukushima, Japan; 15 Department of Internal Medicine, Kashima Hospital, Fukushima, Japan; 16 Department of Neurosurgery, Fukushima Rosai Hospital, Fukushima, Japan; 17 Futaba Medical Center, Fukushima, Japan; 18 Department of Gastroenterology, Soma General Hospital, Fukushima, Japan; 19 Department of Gastroenterology, Soma General Hospital, Fukushima, Japan; 20 Department of Pulmonary Medicine, Minamisoma Municipal General Hospital, Fukushima, Japan; 21 Department of Surgery, Onahama Chuo Clinic, Fukushima, Japan; 22 Department of General Medicine, Shirakawa Satellite for Teaching and Research, Fukushima Medical University, Fukushima, Japan; 23 The First Department of Internal Medicine, Shirakawa Kosei Hospital, Fukushima, Japan; 24 Department of Cardiology and Vascular Medicine, Hoshi General Hospital, Fukushima, Japan; 25 Department of Internal Medicine, Iwase General Hospital, Fukushima, Japan; 26 Department of Surgery, Southern TOHOKU General Hospital, Fukushima, Japan; 27 Department of Pulmonary Medicine, Jusendo General Hospital, Fukushima, Japan; 28 Department of Emergency and Critical Care Medicine, Ohta Nishinouchi Hospital, Fukushima, Japan; 29 Department of Respiratory Medicine, Tsuboi Hospital, Fukushima, Japan; 30 Wakamatsu Infection Leading Clinic, Fukushima, Japan; 31 Department of Emergency and Critical Care Medicine, Fukushima Medical University, Fukushima, Japan; 32 Department of General Internal Medicine, Fukushima Medical University, Fukushima, Japan

**Keywords:** Molnupiravir, COVID-19, Omicron variant, Real-world, Effectiveness

## Abstract

It is unclear whether molnupiravir has a beneficial effect on vaccinated patients infected with the Omicron variant of severe acute respiratory syndrome coronavirus 2 (SARS-CoV-2). We here evaluated the efficacy of molnupiravir in patients with mild-to-moderate coronavirus disease 2019 (COVID-19) during the Omicron variant surge in Fukushima Prefecture, Japan. We enrolled patients with mild-to-moderate COVID-19 who were admitted to hospitals between January and April, 2022. Clinical deterioration after admission was compared between molnupiravir users (*n* = 230) and non-users (*n*  = 690) after 1:3 propensity score matching. Additionally, we performed forward stepwise multivariate logistic regression analysis to evaluate the association between clinical deterioration after admission and molnupiravir treatment in the 1:3 propensity score-matched subjects. The characteristics of participants in both groups were balanced as indicated by covariates with a standardized mean difference of < 0.1. Regarding comorbidities, there was no imbalance between the two groups, except for the presence of hypertension, dyslipidemia, diabetes mellitus, and cardiac disease. The clinical deterioration rate was significantly lower in the molnupiravir users compared to the non-users (3.90% vs 8.40%; *P* = 0.034). Multivariate logistic regression analysis demonstrated that receiving molnupiravir was a factor for preventing deterioration (odds ratio 0.448; 95% confidence interval 0.206–0.973; *P* = 0.042), independent of other covariates. This real-world study demonstrates that molnupiravir contributes to the prevention of deterioration in COVID-19 patients after hospitalization during the Omicron variant phase.

## Introduction

The coronavirus disease 2019 (COVID-19), which originated in Wuhan, China in 2019, remains a serious concern worldwide. Until now, several neutralizing monoclonal antibody products and antiviral agents against severe acute respiratory syndrome coronavirus 2 (SARS-CoV-2) have been developed and authorized by the United States Food and Drug Administration for treatment of high-risk patients with mild-to-moderate COVID-19 [1–4]. However, these drugs have been authorized based on double-blind, placebo-controlled randomized clinical trials [1–4], including trials that targeted only unvaccinated patients with COVID-19 [3, 4]. Therefore, it is important to evaluate the efficacy of these drugs for cases of COVID-19 in real-world settings, where most people are vaccinated.

Newly emerging variants have mutations in the spike protein of SARS-CoV-2 and show high infectivity [5]. The B.1.1.529 (Omicron) variant of SARS-CoV-2, which was first identified on November 25, 2021, in South Africa [6], has attained global dominance with a higher infectivity, transmissibility and immune evasion [7]. Indeed, cases of the Omicron variant have spread throughout Japan from November 2021 [8].

The Omicron variant contains approximately 30 mutations in the spike protein and, in vitro, escapes some neutralizing monoclonal antibodies [9, 10]. To date, few investigations have reported the real-world effect of molnupiravir and nirmatrelvir/ritonavir on COVID-19 during the Omicron variant phase, and in such an investigation by Wong et al. the rate of vaccination was low [11]. It remains unclear whether molnupiravir has a beneficial effect on vaccinated patients infected with the Omicron variant of SARS-CoV-2, as the vaccine is effective in controlling disease exacerbations.

Starting in March 2020, we have been gathering the medical information of patients with COVID-19, who were hospitalized in 27 medical institutes in Fukushima Prefecture, in our electronic database. A total of 6,657 COVID-19 patients were registered by the end of April 2022. Even in Fukushima Prefecture, the Omicron variant was widely spread, and from January 2022, the most frequently detected variant of SARS-CoV-2 was the Omicron variant [12]. Furthermore, sotrovimab and molnupiravir were approved in Japan, and their administration was started in September 2021 and December 2021, respectively, for treating mild-to-moderate COVID-19 patients who are at high-risk of deterioration. Hence, with the use of our database, it was possible to evaluate the clinical efficacy of the above-mentioned new drugs against SARS-CoV-2 including the Omicron variant in the real-world setting. Molnupiravir can be used for outpatients with COVID-19 because it is an oral antiviral drug and is easier to prescribe than monoclonal antibodies such as sotrovimab. Thus, it is important to evaluate the effectiveness of molnupiravir for the Omicron variant of SARS-CoV-2, considering the status of the current pandemic situation.

To the best of our knowledge, the present study is the first real-world retrospective study to evaluate the efficacy of molnupiravir for mostly vaccinated patients with mild-to-moderate COVID-19 caused by the Omicron variant of SARS-CoV-2. We compared the clinical outcomes of the patients treated with and without molnupiravir.

## Material and methods

### Patient consent statement

The need for informed consent was waived because the study is retrospective. This study was approved by the Ethics Committee of Fukushima Medical University (approval number 2020–118, approved on August 3, 2020, updated September 01, 2021).

### Study design and population

**Fig. 1:**
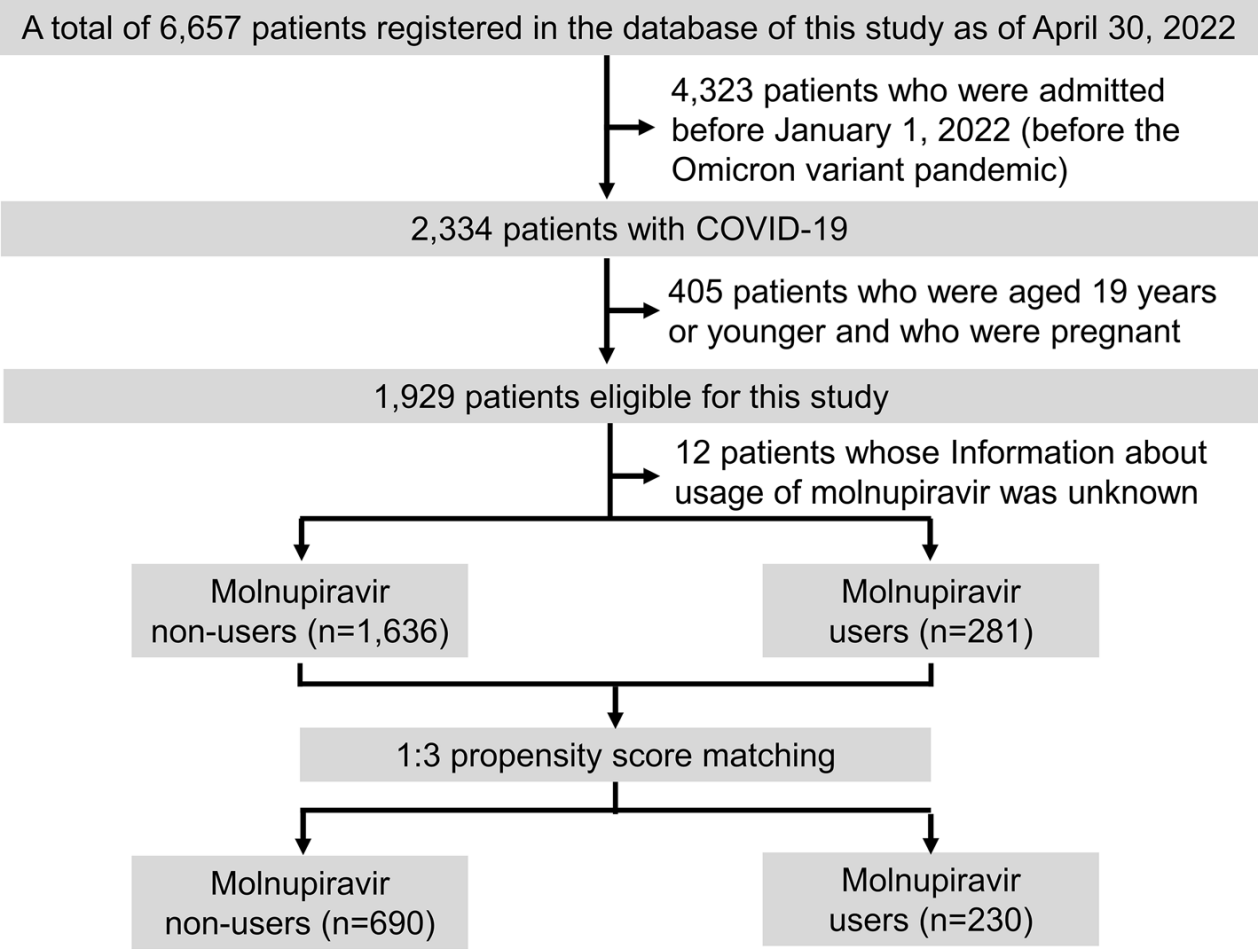
Flowchart of patients’ recruitment in this study. Among a total of 6,657 registered COVID-19 patients in our electronic database, 1,929 were selected for the present study. Eligible participants were matched using 1:3 propensity score matching

This is a retrospective cohort study conducted using an electronic database. Among 27 hospitals participating in this study, we excluded four hospitals whose data on at least 60% of patients were not input into the database. A total of 6,657 COVID-19 patients (as of the end of April 2022) admitted to 23 hospitals in Fukushima Prefecture were enrolled. The 23 hospitals participated in the web conferences organized by the Department of Pulmonary Medicine, Fukushima Medical University. Among the 6,657 patients, the data of 4,323 were excluded, because those patients were admitted before January 1, 2022 (before the Omicron variant pandemic). Among the remaining 2,334 patients, we excluded 405 who were 19 years old or younger and who were pregnant. Finally, we analyzed the data of 1,929 patients, including those who had been vaccinated against SARS-CoV-2. Additionally, we used the propensity score technique to match the molnupiravir users and non-users. Based on the propensity score technique as described in the Statistical analyses section, 230 molnupiravir users were further matched with 690 non-users at a ratio of 1:3 (Fig. 1). The clinical characteristics, including comorbidities, examination results, medications, as well as clinical course and outcomes of the subjects, were obtained from the electronic database of each hospital. Clinical characteristics including severity of all patients were evaluated on the day of admission. The administration of molnupiravir was started on admission in almost all cases.

The diagnosis of COVID-19 was made by positive results for SARS-CoV-2 polymerase chain reaction on nasopharyngeal swab or saliva samples. Assessment of COVID-19 severity was performed according to the definition issued by the Japanese Ministry of Health, Labor and Welfare: mild, patients without pneumonia or respiratory failure; moderate-1, patients with pneumonia but without respiratory failure; moderate-2, patients with pneumonia and respiratory failure (percutaneous oxygen saturation < 94% on room air) but do not require mechanical ventilation/extracorporeal membrane oxygenation (ECMO); or severe, patients with pneumonia and respiratory failure who require mechanical ventilation/ECMO [13, 14]. In addition, we used the DOATS score in order to evaluate the risk of deterioration after admission. The DOATS score is a simple predictive model that we established and reported in a previous study [15]. It consists of four items: 1) having the comorbidity of diabetes or obesity (2 points); 2) age ≥ 40 years (1 point); 3) high body temperature (≥ 38 °C) (1 point); and 4) oxygen saturation < 96% (1 point). The overall score ranges 0 to 5, with a higher score (optimal cutoff point of 2) indicating a higher possibility of deterioration.

Retrospectively, the patients were divided into two groups: (1) those treated with molnupiravir; and (2) those not treated with molnupiravir (controls). If a patient’s condition deteriorated during the clinical course after the administration of molnupiravir, other therapies for COVID-19, including antiviral drugs or immunomodulatory agents (remdesivir, systemic corticosteroid, baricitinib, or tocilizumab), were prescribed and administered at the attending doctor’s discretion.

### Patient eligibility criteria

The inclusion criteria for treatment with molnupiravir were guided by those for the MOVe-OUT trial [3], and the recommendation of the Japanese Ministry of Health, Labor and Welfare [13]. In particular, patients who were aged ≥ 18 years were eligible for molnupiravir treatment if they had symptoms of COVID-19 (e.g., cough, sore throat, fever, and constitutional symptoms), and were within 5 days of symptom onset, and had at least one of the following criteria for high-risk aggravation: an age of > 60 years, body mass index of ≥ 30 kg/m^2^, active cancer, chronic kidney disease (CKD), chronic obstructive pulmonary disease, and presence of serious cardiovascular disease (such as heart failure and coronary artery disease), diabetes mellitus and/or chronic liver disease.

### Outcomes of interest

The primary outcomes of interest were any clinical deterioration, need for mechanical ventilation, and all-cause death after initiation of molnupiravir. The secondary outcomes included the association between treatments and clinical deterioration after hospitalization.

The definition of clinical deterioration in the present study was a worsened respiratory condition requiring additional medications such as systemic corticosteroid, tocilizumab, and baricitinib, or that requiring respiratory therapy (use of inhalation oxygen or mechanical ventilation) after the first day of hospitalization.

### Statistical analyses

Continuous variables are shown as median with interquartile range, and they are shown as mean ± standard deviation when approximately normally distributed. Categorical variables are shown as numbers and percentages. Comparisons between groups for the continuous variables and categorical variables were performed using Mann–Whitney U test and Chi-square test, respectively. Among the comorbidities, those with a prevalence of ≥ 2% were applied to the analyses.

Propensity scores for each subject were calculated via multivariate logistic regression analysis exploring the association between treatment with molnupiravir and other clinical covariates which may influence the treatment selection, such as age, gender, vaccination status, disease severity, DOATS score, pneumonia diagnosed by CT scan, chronic respiratory disease, malignancy, need for nursing care, and chronic kidney disease. In order to select matched controls, a 1:3 propensity score matching method was performed using the nearest neighbor method with a caliper width equal to 0.2 of the standard deviation of logit of the propensity scores [16]. The covariate balance in the baseline characteristics obtained by propensity score matching was assessed by calculating a standardized mean difference (SMD) for each covariate using a matched sample. The cutoff value of the SMD representing imbalance was set at 0.1 [17].

Furthermore, forward stepwise multivariate logistic regression analysis exploring the association between clinical deterioration after hospitalization and treatment with molnupiravir was performed while adjusting for all other covariates in the 1:3 propensity score-matched subjects. Akaike Information Criteria parameters were used to choose the best-fitted model.

All statistical analyses were performed using JMP 13 (SAS Institute Inc, Cary NC) and EZR (Saitama Medical Center, Jichi Medical University, Saitama, Japan). A two-tailed *P* value of < 0.05 was considered statistically significant.

## Results

### Characteristics of participants before and after propensity score matching

The patient selection flowchart is shown in Fig. 1. Among the 1,929 COVID-19 patients enrolled in the current study, 281 were administered molnupiravir. The use of molnupiravir was unknown in 12 subjects due to missing data that had not been input by researchers. The differences in characteristics before matching between the molnupiravir users and non-users are shown in Table 1.

**Table1:** Basic clinical characteristics between the molnupiravir users and non-users before and after 1:3 propensity score matching

Clinical covariates	After matching			Before matching		
	Molnupiravir non-users (*n* = 690)	Molnupiravir users (*n* = 230)	SMD	Molnupiravir non-users (*n* = 1636)	Molnupiravir users (*n* = 281)	SMD
Age, years	64.7 ± 20.1	64.1 ± 20.0	0.034	58.6 ± 22.5	66.0 ± 19.3	0.352
Male sex	353 (51.2)	122 (53.0)	0.038	789 (48.3)	150 (53.6)	0.106
Current smoker	116 (17.1)	38 (16.7)	0.136	290 (18.1)	44 (16.0)	0.079
Received vaccine twice or more	564 (81.7)	189 (82.2)	0.011	1260 (77.0)	220 (78.3)	0.058
Severity, Mild/Mod-1/Mod-2/Severe	556/111/22/1	181/46/2/1	0.197	1113/352/151/10	225/52/2/2	0.422
DOATS score	2 [1, 3]	2 [1, 3]	0.025	1 [1, 2]	2 [1, 3]	0.265
Pneumonia diagnosed by CT scan	131 (21.0)	47 (20.8)	0.006	487 (33.8)	58 (21.2)	0.285
Chronic respiratory disease	82 (11.9)	29 (12.6)	0.022	166 (11.1)	42 (15.7)	0.135
Chronic kidney disease	47 (6.80)	18 (7.80)	0.039	89 (6.00)	23 (8.70)	0.104
Need for nursing care/Bedridden	58 (8.40)	21 (9.10)	0.026	122 (8.10)	27 (10.2)	0.070
Malignancies	48 (7.00)	15 (6.50)	0.017	95 (6.40)	26 (9.60)	0.120
Hypertension	269 (39.0)	126 (55.3)	0.329	509 (33.6)	154 (57.0)	0.485
Dyslipidemia	77 (11.2)	48 (20.9)	0.267	156 (10.5)	56 (21.1)	0.292
Diabetes mellitus	139 (20.1)	60 (26.1)	0.141	262 (17.5)	76 (28.3)	0.257
Obesity	109 (15.8)	38 (16.5)	0.020	190 (12.8)	42 (15.8)	0.086
Cardiac disease	100 (14.5)	42 (18.3)	0.101	199 (13.3)	55 (20.5)	0.193
Stroke	52 (7.50)	13 (5.70)	0.075	96 (6.40)	18 (6.70)	0.013
Autoimmune disease	18 (2.60)	9 (3.90)	0.073	40 (2.70)	11 (4.10)	0.078
WBC, /uL	5541 ± 2195	5357 ± 2359	0.081	5600 ± 2649	5589 ± 4860	0.003
Neutrophil, %	63.5 ± 13.4	62.4 ± 13.6	0.077	63.6 ± 13.8	62.6 ± 13.4	0.074
Lymphocyte, %	25.6 ± 12.0	25.0 ± 12.2	0.049	25.4 ± 12.1	24.6 ± 11.9	0.061
LDH, IU/L	192 [168, 226]	190 [168, 214]	0.179	191 [165, 225]	191 [168, 217]	0.060
CRP, mg/dL	0.92 [0.36, 2.22]	0.94 [0.44, 2.45]	0.046	0.97 [0.34, 2.52]	0.98 [0.40, 2.45]	0.013
Sotrovimab	58 (8.40)	9 (3.90)	0.387	494 (30.2)	169 (60.1)	0.631

Information about usage of molnupiravir was not available in 12 subjects before matching group

Continuous variables are shown as medians with interquartile range except for age, WBC, neutrophil, and lymphocyte. Age, WBC, neutrophil, and lymphocyte are shown as mean ± standard deviation. Categorical variables are shown as numbers with percentages. COVID-19 severity grade: mild, subjects without pneumonia or respiratory failure; moderate-1, subjects with pneumonia but without having respiratory failure; moderate-2, subjects with pneumonia and respiratory failure (oxygen saturation < 94% on room air) but who do not require mechanical ventilation/extracorporeal membrane oxygenation (ECMO); and severe, subjects with pneumonia and respiratory failure who require mechanical ventilation/ECMO

Definition of abbreviations: CRP, C-reactive protein; CT, computed tomography; DOATS score, a predictive model for clinical deterioration in mild-to-moderate COVID-19 patients using four items, having diabetes or obesity, age ≥ 40 years, high body temperature (≥ 38 °C) and oxygen saturation < 96% (Details are described in the manuscript); LDH, lactate dehydrogenase; Mod-1, moderate-1; Mod-2, Moderate-2; SMD, standardized mean difference; WBC, white blood cell

Using the 1:3 propensity score matching method, 230 patients treated with molnupiravir and 690 control patients who did not receive molnupiravir were selected. The baseline clinical characteristics after adjusting for propensity score are summarized in Table 1. After matching, the characteristics of participants in both groups were balanced as indicated by covariates with SMD < 0.1. In the post-propensity score-matched molnupiravir user group, the median age was 64.1 years, 53.0% were male, and 16.7% were current smokers. Regarding comorbidities, there was no imbalance between the two groups (SMD < 0.1), except for the presence of hypertension, dyslipidemia, diabetes mellitus, and cardiac disease. However, those *P* values in Chi-square test were < 0.001, < 0.001, 0.071, and 0.209, respectively. In the molnupiravir user group, the median vaccination rate was 82.2% and DOATS score was 2, which were balanced between the two groups (SMD < 0.1). SMD regarding current smokers and the disease severity were 0.136 and 0.197, respectively, with *P* values in Chi-square test of 0.398 and 0.119, respectively.

### Clinical deterioration rate in molnupiravir users and non-users after 1:3 propensity score matching

The clinical deterioration rate was significantly lower in the molnupiravir users compared to the non-users (3.90% vs 8.40%; *P* = 0.034). One of the non-users required mechanical ventilation, which, however, showed no significant difference between the two groups. Three molnupiravir non-users and two molnupiravir users died, and there was no significant difference regarding death rate between the two groups (Table 2). Univariate logistic regression analysis of deterioration after hospitalization demonstrated that receiving molnupiravir was an independent factor for preventing deterioration (odds ratio [OR] 0.444; 95% confidence interval [CI] 0.216–0.910; *P* = 0.026).

**Table 2:** Comparison of the clinical outcomes between the molnupiravir users and non-users after adjustment with 1:3 propensity score

	Molnupiravir non-user	Molnupiravir user	*P* value	SMD
	*n* = 690	*n* = 230		
Any deterioration	58 (8.40)	9 (3.90)	0.034	0.188
Mechanical ventilation	1 (0.10)	0 (0.00)	1.000	0.054
Death	3 (0.41)	2 (0.90)	0.796	0.054

Definition of abbreviations: SMD, standardized mean difference

### Independent risk factors of deterioration after hospitalization

The results of multivariate logistic regression analysis of the association of COVID-19 deterioration during hospitalization are shown in Table 3. According to this analysis, not receiving molnupiravir was a risk factor related to the clinical deterioration of COVID-19 (OR 0.448; 95% CI 0.206–0.973; *P* = 0.042), independent of other covariates, including the use of sotrovimab. The prevalence of obesity (OR 2.640; 95% CI 1.042–6.687; *P* = 0.041), need for nursing care/bedridden (OR 3.533; 95% CI 1.572–7.940; *P* = 0.002), pneumonia diagnosed by CT scan (OR 2.028; 95% CI 1.087–3.781; *P* = 0.026), high lactate dehydrogenase (LDH) (OR 1.006; 95% CI 1.003–1.009; *P* = 0.000), and high DOATS score (OR 1.847; 95% CI 1.434–2.387; *P* < 0.0001) were also independent risk factors of deterioration after hospitalization.

**Table 3:** Multivariate logistic regression analysis of deterioration after hospitalization among patients with COVID-19 matched using a 1:3 propensity score matching*

	OR	95% CI	*P* value
Received vaccine twice or more	0.581	0.293–1.153	0.121
Hypertension	1.735	0.951–3.168	0.073
Obesity	2.640	1.042–6.687	0.041
Malignancies	6.394	0.781–52.358	0.084
Need for nursing care/Bedridden	3.533	1.572–7.940	0.002
DOATS score, per 1 point-increase	1.847	1.434–2.378	< 0.0001
LDH, per 1 IU/L increase	1.006	1.003–1.009	0.000
Pneumonia diagnosed by CT scan	2.028	1.087–3.781	0.026
Molnupiravir	0.448	0.206–0.973	0.042

^*^Adjusted for age, gender, disease severity, vaccination status, smoking, hypertension, diabetes mellitus, obesity, chronic respiratory disease, malignancies, autoimmune disease, dyslipidemia, cardiac disease, stroke, need for nursing care/bedridden, chronic kidney disease, DOATS score, WBC, neutrophil, lymphocyte, LDH, CRP, pneumonia diagnosed by CT scan, and sotrovimab

COVID-19 severity grade: mild, subjects without pneumonia or respiratory failure; moderate-1, subjects with pneumonia but without having respiratory failure; moderate-2, subjects with pneumonia and respiratory failure (oxygen saturation < 94% on room air) but who do not require mechanical ventilation/extracorporeal membrane oxygenation (ECMO); and severe, subjects with pneumonia and respiratory failure who require mechanical ventilation/ECMO

Definition of abbreviations: CI, confidence interval; CRP, C-reactive protein; CT, computed tomography; DOATS score, a predictive model for clinical deterioration in mild-to-moderate COVID-19 patients using four items, having diabetes or obesity, age ≥ 40 years, high body temperature (≥ 38 °C) and oxygen saturation < 96%; LDH, lactate dehydrogenase; OR, odds ratio. WBC, white blood cell

## Discussion

In the present study, we utilized real-world data from 1,929 COVID-19 patients admitted to hospitals in Fukushima Prefecture between January 2022 and April 2022, during the Omicron variant pandemic, in order to investigate the efficacy of molnupiravir. We performed the 1:3 propensity score matching method, and it is commonly thought that covariates with SMD < 0.25 and < 0.10 are considered moderately balanced and highly balanced, respectively [16]. Therefore, the characteristics of the propensity score-matched participants in both groups were thought to be acceptable in balance including the DOATS score [15], which is a predictor of COVID-19 exacerbation. Hypertension and dyslipidemia are reported to be risk factors of COVID-19 in previous studies [18, 19]. Despite the higher rate of hypertension and dyslipidemia, deterioration rate after hospitalization was significant lower in the molnupiravir users than in the non-users among 1:3 propensity score matching population (Table 2). In addition, we also performed forward stepwise multivariate logistic regression analysis to evaluate the association between the clinical deterioration after hospitalization and the treatment with molnupiravir while adjusting for all other covariates in the 1:3 propensity score-matched subjects. The results showed that not taking molnupiravir was an independent risk factor for deterioration of COVID-19. These results are similar to a previous clinical trial that showed that molnupiravir treatment was associated with significant reductions in hospitalization as well as in the mortality of non-hospitalized patients who were at high risk of mild-to-moderate COVID-19 [3]. Furthermore, in the present study, the patients with a relatively high DOATS score were selected as a result of the 1:3 propensity score matching method. The DOATS score of characteristics between the two groups after 1:3 propensity score matching was well-balanced (the median = 2, SMD = 0.025). Previously, we reported that patients with a high DOATS score (optimal cutoff point of 2) who do not have respiratory failure on admission have a higher possibility of deterioration after hospitalization [15]. Indeed, in the present study, the results of forward stepwise multivariate logistic regression analysis demonstrated that a high DOATS score was an independent risk factor of deterioration after hospitalization (Table 3). Therefore, in the present study, the result that taking molnupiravir was found to be effective in the subjects with the above-mentioned DOATS score strongly supports the clinical significance of molnupiravir administration for patients with COVID-19.

In the preliminary analyses, we also performed 1:1, 1:2, and 1:4 propensity score matching tests, in addition to 1:3 matching. The deterioration rates after hospitalization were significantly lower in the molnupiravir users compared with the non-users (non-users vs. users: 7.5% vs.3.9% in 1:1 matching [*P* = 0.021], 7.9% vs.3.7% in 1:2 matching [*P* = 0.040], and 7.9% vs.3.4% in 1:4 matching [*P* = 0.035]). The number of molnupiravir users was largely reduced in 1:4 matching (*n* = 206). Recently, inverse probability of treatment weighting (IPTW) using propensity score has been used instead of propensity score matching test [20]. IPTW is thought to be able to eliminate background imbalance without losing the size of the study population. We preliminarily tried IPTW in order to adjust the balance of covariates between the molnupiravir users and non-users. However, the disease severity was significantly imbalanced between the two groups in the IPTW method (SMD = 0.415, *P* = 0.001). Therefore, we employed a 1:3 propensity score matching method in the present study, because the number of matched molnupiravir-treated subjects was not so reduced and the number of matched controls was increased (Table 1).

Detailed information about the SARS-CoV-2 variants of individual cases was not available in the current study. However, the Fukushima Prefectural Institute of Public Health reported that the proportion of Omicron variant cases reached 70% by the beginning of January 2022, and reached 100% after mid-February [12]. Therefore, we believe that almost all cases analyzed in this study were the Omicron variant. Therefore, the present study supports the efficacy of molnupiravir against SARS-CoV-2 Omicron variants.

In vitro and in vivo, molnupiravir retains antiviral potency against SARS-CoV-2 variants including B.1.1.529 (Omicron) [21, 22], B.1.1.7 (Alpha), B.1.351 (Beta), B.1.617.2 (Delta), and P.1 (Gamma), and may prevent the selection of drug-resistant variants [23, 24]. Recently, Wong et al. [11]. reported in a retrospective cohort study in Hong Kong that the use of molnupiravir or nirmatrelvir/ritonavir for treating SARS-CoV-2 patients infected with the Omicron variant reduced the all-cause mortality rate along with reduced viral loads compared with control groups (not receiving molnupiravir or nirmatrelvir/ritonaviroral). Although their results support the present study results, the details regarding the disease severity, comorbidity and deterioration rate in the patients treated with molnupiravir in their study are unknown. In addition, the rate of vaccination in their study subjects treated with molnupiravir after propensity score matching is low (about 6%). The vaccination rate of the subjects in our study was high (about 80%), and we demonstrated that molnupiravir is effective for preventing deterioration independently of vaccination. Taking our real-world results together with those from Wong et al., molnupiravior is suggested to be effective for the treatment of the SARS-CoV-2 Omicron variant.

The strength of the current study is that its results are considered to be highly reliable because the population was comprised of inpatients at major 23 institutions in Fukushima Prefecture that handle COVID-19 inpatient treatment. In addition, this retrospective cohort study analyzed the population with a high vaccination rate. Therefore, our study reflects the current real world of the COVID-19 pandemic.

There are several limitations to the present study. First, this was an observational and retrospective study. Therefore, the results of this study cannot be equated with those obtained from a randomized control trial. Furthermore, clinical deterioration, which was set as the primary outcome in this study, is less objective than hard endpoints such as mechanical ventilation and death. However, it is essential to evaluate clinical deterioration from various perspectives, such as individual disease burden, concerns about post-COVID-19 condition, and health economics. Second, the information about the duration between COVID-19 symptom onset and the administration of molnupiravir and the day of exacerbation was not available in our database. The difference in clinical time course may influence the clinical outcomes. Third, we could not assess whether molnupiravir contributes to shortening the length of hospital stay due to the lack of information. Fourth, regarding the vaccinated patients, it was unknown on what day after the vaccination was completed that the inoculator was infected. It can be estimated that almost all healthy individuals obtained the full efficacy of vaccination against SARS-CoV-2 at least 7 days after second vaccination [25]. Some subjects may have been infected with the virus shortly after their injection before acquiring immunity against the virus.

In conclusion, this real-world retrospective study of high-risk mild-to-moderate COVID-19 patients, who had a high vaccination rate, during the Omicron variant pandemic demonstrated a low rate of clinical deterioration after treatment with molnupiravir. Treatment with molnupiravir should be considered to prevent deterioration in high-risk patients with mild-to-moderate COVID-19.

## Data Availability

The datasets generated during and/or analyzed during the current study are not publicly available due to our institutional policy but are available from the corresponding author on reasonable request.

## Abbreviations

CI: Confidence interval
CKD: Chronic kidney disease
COVID-19: Coronavirus disease 2019
CRP: C-reactive protein
CT: Computed tomography
ECMO: Extracorporeal membrane oxygenation
IPTW: Inverse probability of treatment weighting
LDH: Lactate dehydrogenase
OR: Odds ratio
SARS-CoV-2: Severe acute respiratory syndrome coronavirus 2
SMD: Standardized mean difference
